# High resolution HLA haplotyping by imputation for a British population bioresource

**DOI:** 10.1016/j.humimm.2017.01.006

**Published:** 2017-03

**Authors:** Matt J. Neville, Wanseon Lee, Peter Humburg, Daniel Wong, Martin Barnardo, Fredrik Karpe, Julian C. Knight

**Affiliations:** a Oxford Centre for Diabetes, Endocrinology and Metabolism, University of Oxford, Churchill Hospital, Old Road, Oxford OX3 7LJ, UK; b Oxford NIHR Biomedical Research Centre, Churchill Hospital, Oxford, UK; c Wellcome Trust Centre for Human Genetics, University of Oxford, Roosevelt Drive, Oxford OX3 7BN, UK; d Transplant Immunology and Immunogenetics Laboratory, Oxford Transplant Centre, Churchill Hospital, Oxford OX3 7LJ, UK

**Keywords:** AFND, Allele Frequencies Net Database, LD, linkage disequilibrium, MAF, minor allele frequency, OBB, Oxford Biobank, PCA, principal components analysis, SNP, single nucleotide polymorphism, HLA, Allele, Imputation, Genotype, Single nucleotide polymorphism

## Abstract

This study aimed to establish the occurrence and frequency of HLA alleles and haplotypes for a healthy British Caucasian population bioresource from Oxfordshire. We present the results of imputation from HLA SNP genotyping data using SNP2HLA for 5553 individuals from Oxford Biobank, defining one- and two-field alleles together with amino acid polymorphisms. We show that this achieves a high level of accuracy with validation using sequence-specific primer amplification PCR. We define six- and eight-locus HLA haplotypes for this population by Bayesian methods implemented using PHASE. We determine patterns of linkage disequilibrium and recombination for these individuals involving classical HLA loci and show how analysis within a haplotype block structure may be more tractable for imputed data. Our findings contribute to knowledge of HLA diversity in healthy populations and further validate future large-scale use of HLA imputation as an informative approach in population bioresources.

## 1 Introduction

The high level of polymorphism involving classical HLA alleles reflects the importance of the encoded molecules in human health and disease, notably in terms of transplantation and autoimmunity but also for diverse phenotypes including drug response and susceptibility to infection [1]. For bone marrow and other donor registries, population level HLA allele frequency data is available for diverse ethnic groups worldwide through the International HLA and Immunogenetics Workshop [2], [3] and Allele Frequencies Net Database (AFND) [4], [5], [6]. These include high resolution HLA haplotype frequencies in US populations for the entire US donor registry [7] and large scale data for German donors [8], [9] while databases of allelic reference sequences and nomenclature are maintained by IPD-IMGT/HLA (http://www.ebi.ac.uk/imgt/hla) [10]. There are a range of methods for direct HLA typing including serological testing, use of sequence-specific amplification primers (SSP) or probes (SSO), Sanger sequencing and next generation sequencing based typing [11], [12]. Imputation of HLA alleles from SNP genotyping [13], [14], [15], [16], [17] provides a further complementary approach of significant interest given the low cost and broad availability of accurate high throughput genotyping through genome-wide association studies and other initiatives. With the high number of disease associations mapping to the MHC and the diverse collections of disease cohorts with high density chip data becoming available, accurate HLA imputation can enhance the informativeness of SNP data significantly [16], [18].

Here, we sought to apply SNP based HLA imputation to a large United Kingdom (UK) Bioresource to add to the existing data on the accuracy and application of the approach, to define HLA allele frequencies for a homogenous health British Caucasian cohort recruited from Oxfordshire UK and understand patterns of haplotypic recombination in this group. Oxford Biobank (OBB) is a bioresource of male and female residents from Oxfordshire used in different studies including the opportunity to recruit-by-genotype and recruit-by-phenotype [19] and is part of the NIHR National Bioresource. Existing British individuals with large-scale HLA typing data include the Welsh bone marrow registry (>21,000 individuals) [20] and the UK renal transplant list (7007 individuals) [21] while the 1958 Birth Cohort (http://www.cls.ioe.ac.uk) has provided both gold-standard two-field typing data for 918 individuals and SNP genotyping. In this paper, we report application of the SNP2HLA methodology [16] to impute HLA alleles and amino acid polymorphisms from dense SNP genotyping data on the OBB cohort with validation using direct typing. The authors of the SNP2HLA software have previously shown that with a suitably large training set high levels of accuracy in HLA imputation can be achieved [16]. This method also adds a further level of information for genetic disease studies by imputing amino acid differences involving classical HLA genes, which is of growing interest given evidence that specific disease associations can be resolved to particular amino acid polymorphisms such as seen in rheumatoid arthritis [22] and psoriasis [23], and is of significant potential value in the setting of bioresource cohorts.

## 2 Materials and methods

### 2.1 Study population

OBB (www.oxfordbiobank.org.uk) was established in 2000 as a random population based cohort of healthy Caucasian men and women aged 30–50 years to enable recruitment of participants into primary and early translational research for the Oxford and UK research community [19]. As of July 2016, 7900 participants have been recruited. The OBB is also part of the UK National NIHR Bioresource (https://bioresource.nihr.ac.uk), a collection of over 100,000 individuals from both control and disease cohorts with consent in place to recall for recruit-by-genotype studies. Extensive screening information is collected on all individuals including: anthropometry, biochemistry, questionnaires and blood pressure. In addition, DXA body composition imaging using Lunar iDXA (GE Healthcare, Lunar, Madison, WI) (n = 5200 participants), NMR based (n = 5500) and Metabolon mass spectroscopy based (n = 2250) metabolomics data have been generated together with SNP genotyping (detailed in Section 2.2) (n = 6000). All individuals have given informed consent to be contacted again at a later date for targeted research studies (COREC reference 08/H0606/107+5).

### 2.2 DNA extraction, genotyping and quality control

DNA was extracted commercially from 8 to 10 ml whole blood and 260/280 nm spectrophotometer ratios generated to assess quality (LGC Genomics, Hoddesdon, UK). Samples were genotyped using the Illumina HumanExome-12v1_A beadchip array (Illumina, San Diego, CA) and variants called using Illumina GenCall algorithm [24] from standard Illumina cluster files. Samples were excluded on call rate <98%, heterozygosity 4SD of mean, exact HWE <10^−4^, and on self-reported non-Caucasian ancestry. The Illumina HumanExome array was designed to facilitate large-scale genotyping of 247,870 mostly rare (minor allele frequency (MAF) <0.5%) and low-frequency (MAF 0.5–5%) protein altering variants selected from sequenced exomes and genomes of ∼12,000 individuals. In addition, a set of 2536 SNPs from within the HLA region of chromosome 6 were included in the design to facilitate future classical HLA type imputation [16].

### 2.3 HLA imputation using SNP2HLA

The SNP2HLA software tool [16] was used to impute one and two field resolution classical HLA alleles and to impute amino acid substitutions identified as a consequence of polymorphic nucleotides for the *HLA-A*, *-C*, *-B*, *-DRB1*, *-DQA1*, *-DQB1*, *-DPA1* and *-DPB1* gene loci within the MHC region on chromosome 6. SNP2HLA_package_v1.0.2 [16], Beagle.3.0.4 [25], linkage2beagle_2.0 [16] and Plink1.07 [26] were used following recommended parameters with 10 iterations and a marker window size of 1000. The pre-built Type 1 Diabetes Genetics Consortium (T1DGC) reference panel of 5225 European individuals and 8961 binary markers was downloaded along with the SNP2HLA tool and used as a training set for the HLA imputation. After quality control and sample exclusions (Section 2.2), the OBB Illumina Exome Chip dataset comprised data for 5553 individuals. A total of 4098 SNP markers between coordinates chr 6:25653609-45095163 (GRCH37/hg19) were extracted using PLINK [26] for HLA imputation. There was an overlap of 1694 markers between the OBB data set and the T1DGC data set. As well as the imputed HLA alleles and amino acids, imputation posterior probabilities were also determined to inform the accuracy of the imputed alleles.

### 2.4 HLA typing using sequence-specific primer amplification

To assess the accuracy of the HLA imputation, intermediate resolution classical HLA class I and II typing of 5 loci (*HLA-A*, *B*, *C*, *DRB1*, *DQB1*) was performed on 70 of the OBB individuals by SSP as previously described [27]. This was carried out in the Transplant Immunology Laboratory at the Oxford Transplant Centre. Intermediate resolution was considered a practical resolution level to compare with imputation. Whilst this resolution does not define the definitive two-field HLA types it does give extra information above one-field to enable groups of alleles to be differentiated into smaller groups that separate common subtypes (eg B*14:01/07N/14/26/32/40/46/47/49/54 can be distinguished from B*14:02/04/09/11/15/16/17/18/20/22/25/29/31/34/35/36/38/39/41N/43/44/45/48/50/51/52).

### 2.5 HLA haplotypes and recombination rate estimation

Linkage disequilibrium (LD) extends across the whole of the MHC with ancestral extended haplotypes spanning *HLA-A* and *HLA-DQB1* defined in a number of populations. Homozygous cell lines have been established for several of these haplotypes from which sequence data has been generated [28], [29], [30]. There is interest in using HLA typing to impute ancestral haplotypes at a population level [7], [8], [31], [32]. To assess such haplotypes in OBB, we applied Bayesian methods implemented with the PHASE V2 software [33], [34] to the two-field resolution SNP2HLA data. For six-locus haplotypes (*HLA-A*, *-C*, *-B*, *-DRB1*, *-DQA1*, *-DQB1*), PHASE was run with 30,000 iterations, a thinning interval of 10 and a burn-in of 100, this took about 4 weeks to run (on iMac 3.4 GHz Intel Core i7 with 32 Gb ram running OSX10.8). For the more complex full eight-locus haplotypes (*HLA-A*, *-C*, *-B*, *-DRB1*, *-DQA1*, *-DQB1*, *-DPA1*, *-DPB1*) the computational time proved to be prohibitively long therefore a reduced number of 1000 iterations was run to generate an estimate, all be it at a reduced accuracy compared to the six-locus haplotypes. As with the SNP2HLA software, confidence probabilities generated by PHASE were also used to assess the certainty of the haplotype being correct. Pairwise LD between specific HLA alleles defined in the most frequent eight-locus haplotypes was calculated in PLINK. To estimate the recombination rate and assess recombination hotspots within the selected HLA region, additional runs were performed in PHASE V2 using the – MR flag to specify the PAC-likelihood recombination rate model [35]. This was run with 1000 iterations and the algorithm was run 5 times using the – x5 flag. The median recombination rate estimates between each locus were calculated from the PHASE_recom output and rescaled to the PHASE calculated background recombination rate. Optimal haplotype blocks were defined based on analysis of recombination rates across the region. Haplotypes were then constructed for these multi-locus haplotype blocks using PHASE V2 with 10,000 iterations.

### 2.6 Principal components analysis

SNPs located in coding regions were used to carry out a principal components analysis (PCA) using the SNPRelate program [36]. The Illumina HumanExome array SNPs for the 5553 OBB individuals were compared to SNP genotypes for 1397 individuals from 11 human populations generated by the HapMap project (phase III) [37]. From the 206526 SNPs in the OBB exome chip data that passed the QC cutoffs and the 1457897 SNPs in HapMap, a total of 20560 SNPs overlapped in both data sets. These were merged using Plink [26]. 146 mis-matching SNPs between the two datasets and 172 SNPs on non-autosomes were additionally removed. SNPs with LD threshold more than 0.2 were excluded from the analyses to avoid the effect of SNP clusters in PCA. After filtering by LD, there were 11780 SNPs available for genome-wide PCA analysis. For PCA restricted to the MHC region, 242 SNPs were used after filtering by LD. Due to the imbalance in number of individuals in different population between the two datasets, we further randomly selected 150 samples from OBB data and performed PCA analysis.

## 3 Results

### 3.1 Demographics and population genetics of study cohort

**Fig. 1:**
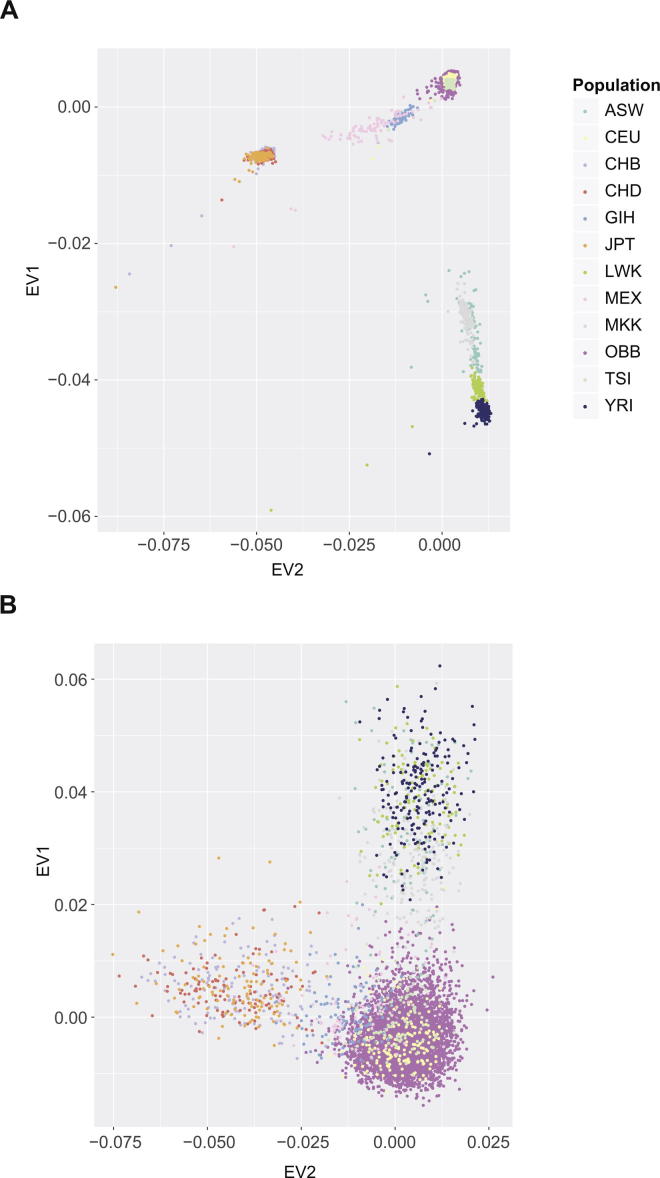
Principal component analysis comparing Oxford Biobank participants with 11 different human populations from the HapMap project. (A) Data is shown for British Caucasian individuals from Oxfordshire participating in OBB (5553 people) and for individuals from 11 HapMap populations (1397 people) using genome-wide SNP typing. First and second principal components shown plotted as eigenvectors (EV). ASW (African ancestry in Southwest USA, n = 87); CEU (Utah residents with Northern and Western European ancestry from the CEPH collection, 165); CHB (Han Chinese in Beijing China, 137); CHD (Chinese in Metropolitan Denver Colorado, 109); GIH (Gujarati Indians in Houston Texas, 101); JPT (Japanese in Tokyo Japan 113); LWK (Luhya in Webuye Kenya 110), MEX (Mexican ancestry in Los Angeles, California 86); MKK (Maasai in Kinyawa Kenya 184); TSI (Toscani in Italia, 102); YRI (Yoruba in Ibadan Nigeria, 203). (B) PCA restricted to SNPs in the MHC region.

High quality genotyping data including 2536 SNPs from the HLA region were available for 5553 individuals following data processing and quality control. These were all healthy adult British volunteers of self-reported Caucasian ancestry living in Oxfordshire UK and recruited to OBB. They comprised 2469 males and 3084 females with a mean age of 41.7 ± 5.8 (males 41.9 ± 5.6, females 41.5 ± 6). To assess the self-reported ancestry of the participants and avoid any population-specific allelic variation in our analysis we first performed PCA analysis comparing SNPs genotyped in both the OBB samples and 11 diverse global populations from the HapMap project (1397 individuals) [37]. This demonstrated clear clustering of all the OBB individuals with CEU individuals of Northern and Western European ancestry (Fig.1A). This was also seen when we restricted the PCA to SNPs in the MHC region (Fig.1B). This showed that all the OBB individuals continued to overlap with the CEU population (Fig.1B). PCA plots using 150 randomly selected individuals from OBB to allow comparison of equivalent sample sizes are shown in Supplementary Fig. 1.

### 3.2 HLA imputation

**Fig. 2:**
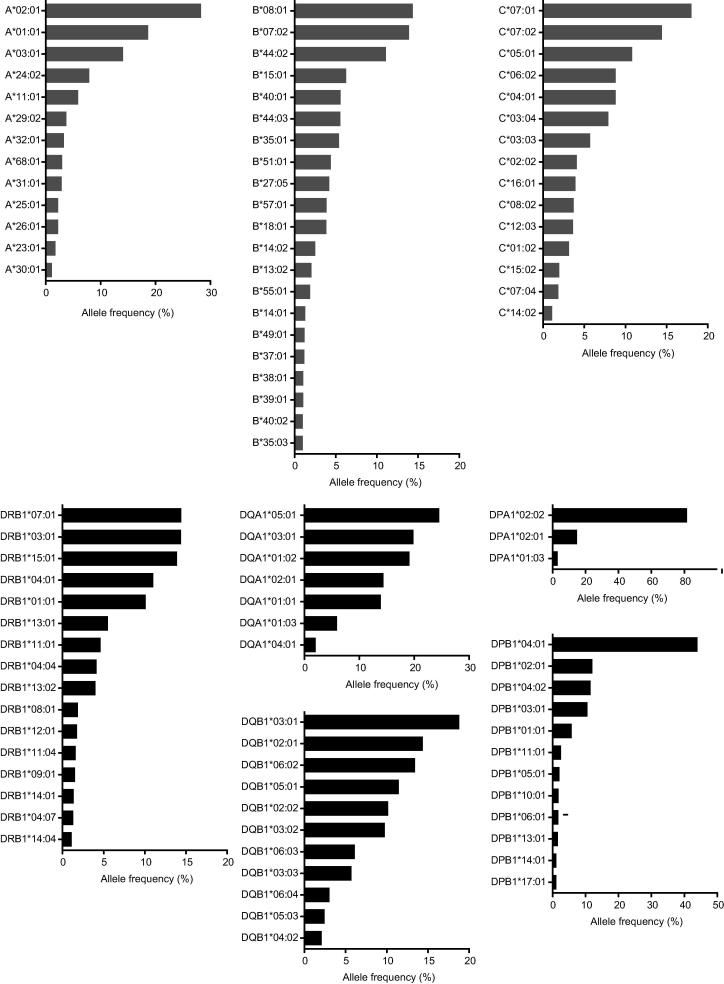
HLA allele frequencies observed in 5553 British Caucasian individuals from Oxfordshire participating in Oxford Biobank. Classical class I and class II allele frequencies shown for 11,106 chromosomes based on imputation. HLA alleles with a frequency of 1% or greater are shown. The full list of alleles identified is shown in Supplementary Table 1A.

Classical HLA alleles were imputed for 8 loci (*HLA-A*, *HLA-B*, *HLA-C*, *HLA-DRB1*, *HLA-DQA1*, *HLA-DQB1*, *HLA-DPA1* and *HLA-DPB1*) using SNP2HLA for 5553 OBB individuals of Caucasian ancestry. A total of 62 one-field and 110 two-field HLA class I alleles (32 *HLA-A*, 56 *HLA-B*, 22 *HLA-C*) were imputed for this population cohort, plus 47 one-field and 85 two-field class II alleles (34 *HLA-DRB1*, 8 *HLA-DQA1*, 16 *HLA-DQB1*, 6 *HLA-DPA1* and 21 *HLA-DPB1*) (Table 1) (Supplementary Table 1A and 1B). The distribution of allele frequencies is illustrated in Fig. 2.

**Fig. 3:**
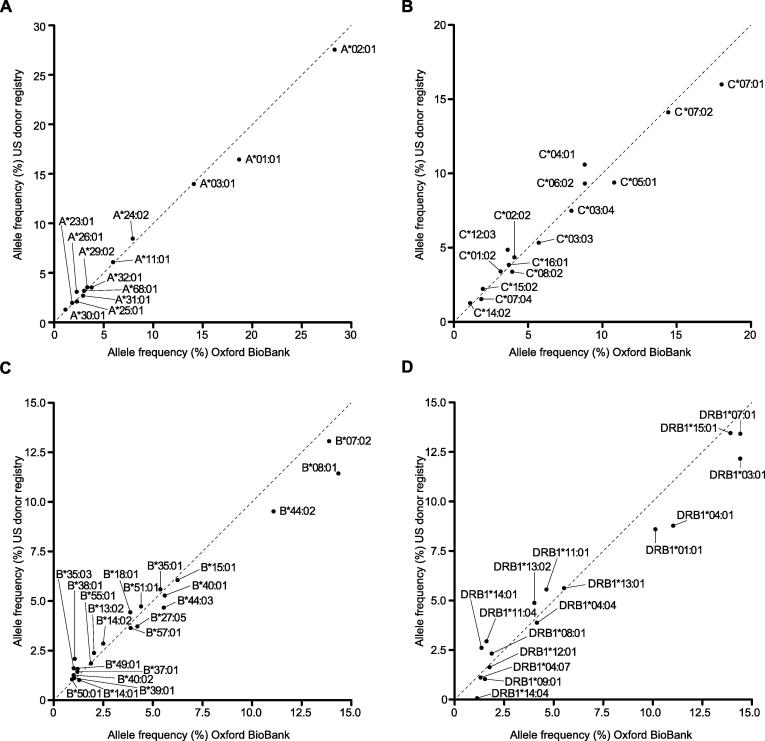
Comparison of imputed HLA allele frequencies between populations. Allele frequencies for classical HLA alleles in British Caucasian individuals from OBB (5553 people) plotted vs available laboratory-typed allele frequencies in individuals of Caucasian ancestry in US donor registry (1,242,890 people) [7]. A high degree of correlation was seen (for alleles with MAF >1%, r^2^ 0.99 for *HLA-A*, 0.98 for *HLA-B*, 0.98 for *HLA-C* and 0.96 for *HLA-DRB1*).

One of the largest published datasets of high resolution HLA types is from the US donor registry, comprising 6.59 million subjects of which 1.24 million are of European Caucasian ancestry [7]. We proceeded to compare the observed imputed allele frequencies in our British Caucasian population from OBB with the US donor data generated from individuals of European Caucasian ancestry. *HLA-A*, *-C*, *-B* and *-DRB1* loci data were available for comparison from the US cohort. The observed allele frequencies for these 4 loci were highly comparable (Fig. 3 and Supplementary Table 1A). Among alleles with MAF >1%, the observed correlation r^2^ was 0.99 for *HLA-A*, 0.98 for *HLA-B*, 0.98 for *HLA -C* and 0.96 for *HLA-DRB1*. Consistent with this, for class I alleles the overall rank order in terms of allele frequency between the populations was very similar, although for *HLA-B* the highest frequency allele in the UK OBB population was HLA-B*08:01 rather than HLA-B*07:02 in the US population (Supplementary Table 1A). For class II alleles, rank order was broadly consistent but greater variation was seen (Supplementary Table 1A).

We next assessed the confidence of imputation based on posterior probabilities for imputed variants. Overall, for alleles with a MAF >5% we found that alleles were imputed with a posterior probability of >0.95 accuracy in over 90% of the individuals. However, we found significant variation between loci, with highest confidence based on this parameter for class I alleles, with HLA-DRB1 and HLA-DPB1 alleles imputed with lower confidence (Table 1) (Supplementary Table 1A).

We also used SNP2HLA to impute amino acid residue substitutions as a consequence of polymorphic SNP loci for this British Caucasian population. Of the combined total of 2393 amino acids across the 8 HLA proteins (refseq counts: *HLA-A*_365aa, *-C*_366aa, *-B*_362aa, *-DRB1*_266, *-DQA1*_255aa, *-DQB1*_261aa, *-DPA1*_260 and *-DPB1*_258aa) a total of 393 polymorphic amino acid positions were imputed, of which 214 (54.5%) were biallelic and 179 (45.5%) were multi-allelic (Table 2). From these 393 positions a total of 1108 alternate amino acid residues were observed in this population, with highest numbers of alternate amino acid residues seen for *HLA-B* and *HLA-DRB1* (Table 2 and Supplementary Table 2).

### 3.3 Validation

To validate the imputed HLA alleles, 70 OBB individuals (140 chromosomes) were directly HLA typed by the SSP method [27] in an ISO15189:2012 and European Federation for Immunogenetics accredited H&I laboratory. For sequence-specific amplification we used forward and reverse allele specific primers in multiple PCR reactions to allow discrimination of *cis* from *trans* alleles across each genomic region, and thus definitively assign HLA types to both homozygous and heterozygous individuals. HLA types for the 5 loci *HLA-A*, *-C*, *-B*, *-DRB1* and *-DQB1* were included in the SSP typing as the minimum required for solid organ and stem cell transplantation in the UK.

Intermediate scale resolution clinical HLA typing is more detailed than the imputed two-field alleles we had established from SNP genotyping, which give a more precise two-field designation but with lower certainty. This is reflected in the greater number of potential allele subtypes grouped together by the clinical typing method (see Section 2.4 and Supplementary Table 3A). The clinical types were compressed into equivalent two-field and one-field resolution HLA types. Among the 70 individuals we found a very high degree of concordance between imputed and SSP typing. The 5 loci typed across the 140 chromosomes represent a total of 700 chromosomal segments. For alleles imputed at two-field resolution only 1% were discordant with SSP typing, whilst for the one-field HLA typing 0.3% were discordant (Supplementary Table 3B). Relating this back to the 70 individuals, this represented 6 out of 140 chromosomes discordant at the two-field resolution (4%). Only one individual was discordant for more than one locus (two loci: *HLA-A* and *HLA-C*) and cross-referencing this against the inferred extended haplotypes showed both discordant HLA alleles fell on the same predicted extended HLA haplotype.

### 3.4 Six- and eight-locus resolution HLA haplotypes

**Fig. 4:**
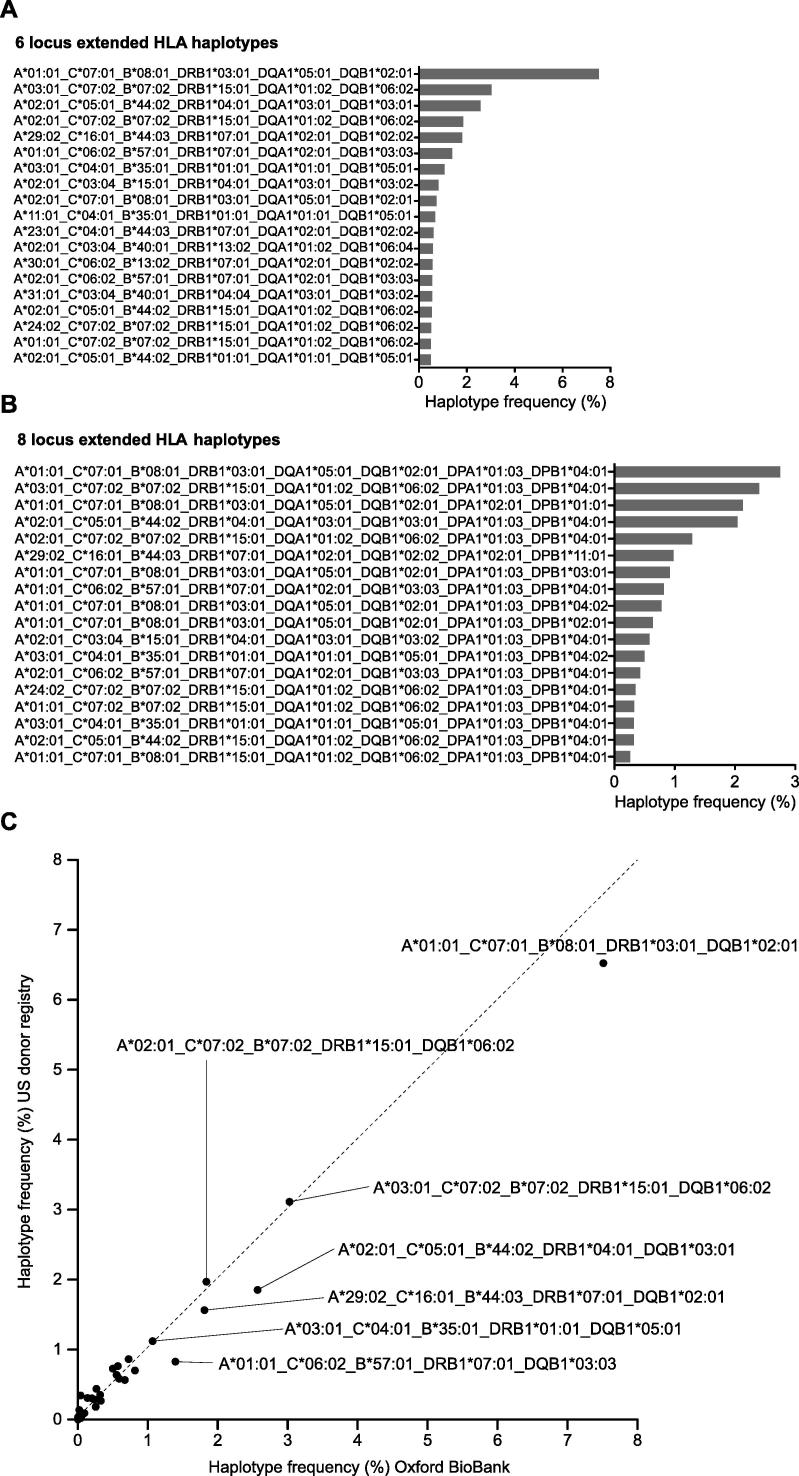
Six- and eight-locus HLA haplotypes in OBB population. The frequencies of the most common observed HLA haplotypes are shown at (A) six-locus (haplotype frequency cutoff of 0.5%) and (B) eight-locus resolution (haplotype frequency cutoff of 0.25%). (C) Comparison of haplotype frequencies in British Caucasian individuals from OBB plotted vs individuals of Caucasian ancestry in US donor registry [7].

**Fig. 5:**
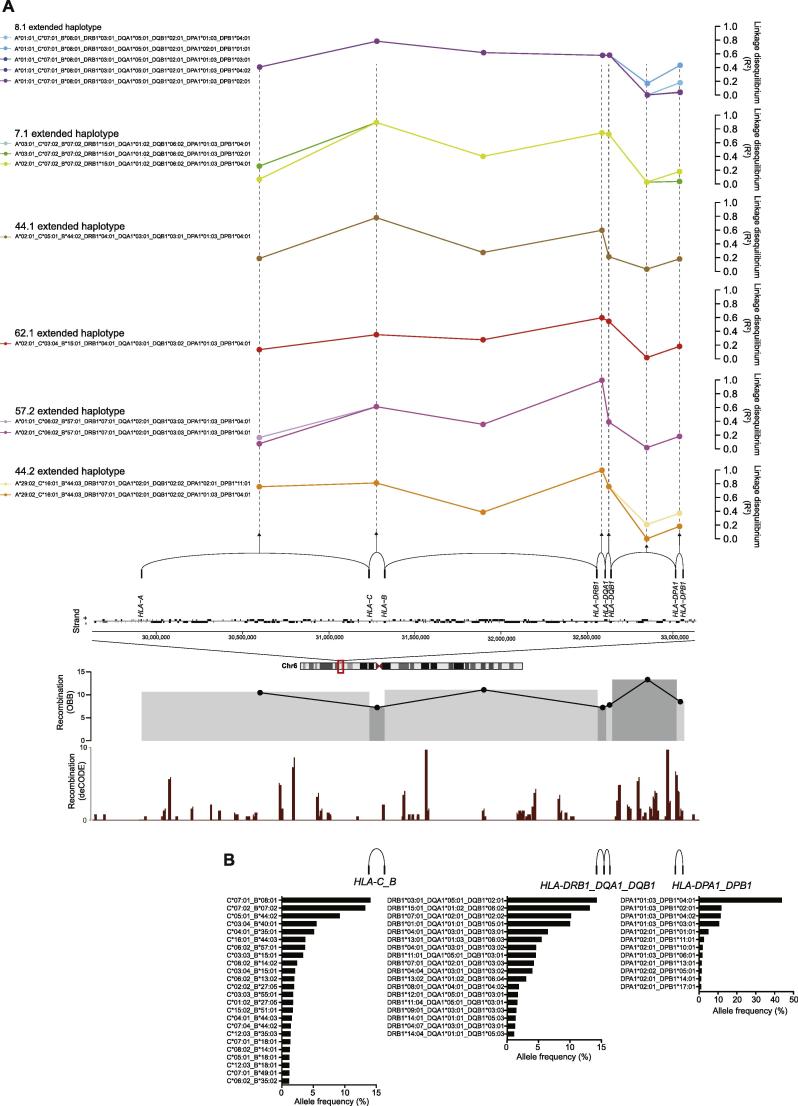
Linkage disequilibrium and extended HLA haplotypes. (A) Pairwise LD (R^2^) between alleles for common 8 loci extended haplotypes observed in OBB population are shown in relation to genomic location (schematic representation) in the MHC region. Below this recombination rate shown for OBB individuals (n = 5544) (grey shaded blocks) calculated using PHASE with log median recombination rescaled to the background recombination rage of 1 plotted on the y axis; and data from the recombination generated by deCODE [54] showing calculated rates of recombination (sex-averaged) using 10-kb windows. (B) Allele frequencies for haplotype blocks spanning low recombination regions with data for HLA-C/HLA-B alleles, HLA-DRB1/-DQA1/-DQB1 alleles and HLA-DPA1/DPB1 shown where observed haplotypes >1% frequency.

We proceeded to investigate the occurrence of HLA haplotypes in this British Caucasian population. Haplotypes were constructed for six- (*HLA-A*, *HLA-C*, *HLA-B*, *HLA-DRB1*, *HLA-DQA1* and *HLA-DQB1*) and eight-locus (including *HLA-DPA1* and *HLA–DPB1*) regions using PHASE from the SNP2HLA imputed alleles and involving 11,088 chromosomes (Supplementary Tables 4A and 4B). The most frequent haplotypes are shown (Fig. 4). We found high concordance for six-locus haplotype frequencies with US donors of European Caucasian ancestry (correlation r^2^ 0.98) (Fig. 4). As expected, the most common observed six-locus haplotype was the 8.1 (COX) ancestral haplotype HLA-A*01:01-C*07:01-B*08:01-DRB1*03:01-DQA1*05:01-DQB1*02:01 which we observed in 7.5% of chromosomes (Fig. 4). Overall, 55 individuals were homozygous for six-locus haplotypes including 28 individuals for AH 8.1 (COX), 5 for AH 44.1 (AWELLS), 5 for AH7.1 (PGF), 3 for AH 44.2(MANN), 1 for AH 60.1(MT14B) and 1 for AH 60.3(EMJ) (Supplementary Table 4A). As others have found [7], [8], [31], [32] the construction of the six-locus haplotypes proved computationally very intensive, primarily due to uncertainties in phase caused by recombination hotspots (see Section 3.5 below). This was especially the case for the 8 locus haplotypes that had an additional recombination hotspot between *HLA-DQB1*and *HLA-DPA1* (Fig.5A). For this reason, although the population level haplotype frequencies were largely similar between our data and the US donor registry, at the individual level the proportion of individuals with a high degree of certainty were low and the number of predicted haplotypes consequently very large. This would be especially the case for rare haplotypes. For the 2488 different six-locus haplotypes we defined, only 52.4% of individuals were assigned with >95% certainty while for eight-locus haplotypes this dropped to 24.3%. It is important to note that all methods of computationally imputing extended haplotypes across this region will have the same problem, although the low degree of certainty for individual level data is rarely discussed.

### 3.5 Haplotype blocks

The MHC region shows complex LD [38], [39] with polymorphic frozen haplotype blocks proposed [40]. Multiple recombination hot spots have been defined [41], [42] together with high resolution LD maps [43]. Non-uniform patterns of LD include regions such as between *HLA-B* and *HLA-C* or *HLA-DRB1* and *HLA-DQA1* where high LD and low recombination are seen. Due to the uncertainties inherent in constructing extended haplotype across the whole region, as discussed in Section 3.4 above, we investigated the utility of haplotype block structure to reduce computational complexity and time and increase certainty, which is particularly pertinent for eight-locus haplotype generation, as discussed above. We estimated recombination rates between classical HLA class I and class II genes in our data set (Fig.5A). Taken with publicly available recombination data, we then defined and constructed haplotypes for three regions of high LD (spanning *HLA-C_B*, *HLA-DRB1_DQA1_DQB1* and *HLA-DPA1_DPB1*) within which we constructed 220, 94 and 39 high confidence haplotypes respectively using PHASE (98.8, 99.8 and 99.5% of individuals assigned with >95% certainty) (Fig.5B) (Supplementary Table 4C). This was a significant improvement on the low certainty attained when taking the whole region together. To further characterize the differences in LD pattern between the ancestral and the extended haplotypes we also calculated pairwise LD between alleles involved in the most common observed eight-locus haplotypes for our OBB population (Fig.5A).

## 4 Discussion

We have presented data that define the HLA allelic landscape for a healthy British Caucasian population in a geographically discrete area of southern England. This provides a resource for future population genetic studies, complementing those available for other cohorts which typically arise from donor registries or patient groups [7], [9], [20], [32]. Our study population involves a bioresource for which knowledge of HLA alleles is of direct utility, with the ability to recall by genotype or phenotype enabling, for example, functional studies of individuals with specific alleles. The successful application of HLA imputation to the large numbers of individuals typically recruited to such bioresources is of significant practical relevance as national scale bioresources are being assembled such as the UK NIHR BioResource (www.bioresource.nihr.ac.uk) and prospective longitudinal cohorts with linked disease incidence/phenotyping such as the Precision Medicine Initiative Cohort Program in the United States (www.nih.gov/precision-medicine-initiative-cohort-program) and UK BioBank (www.ukbiobank.ac.uk). We find that SNP2HLA generated high confidence imputation at one- and two-field resolution which was validated by SSP-based direct HLA typing for 5 loci. Imputation of HLA alleles and amino acid polymorphisms using SNP2HLA has been successfully implemented for genetic studies of associations in a range of traits [44], [45], [46], [47], [48].

A further question arises as to whether HLA haplotypes spanning classical alleles can be generated from such data. Our study shows that this is difficult to achieve for individual level data given current computations tools largely due to the complexity as a result of recombination hotspots. We did find a high level of correlation to that seen in the US donor population [7], however, there was a relatively low certainty attached to individual level data, especially for the rarer haplotypes and for eight-locus HLA haplotype imputation. This reflects both the limitations of current tools as well as the complexity of regional haplotype structure. As one approach to address this, we have presented an analysis of patterns of linkage and recombination across the MHC for HLA haplotypes in our British Caucasian population and the potential utility of defining haplotypes within specific haplotype blocks for individuals when using imputed HLA data. Achieving high confidence imputation for more limited haplotype blocks may be a realistic compromise in work where accurate calling is needed for disease association and mapping studies.

Clinical typing by methods such as SSP are still considered the gold-standard for transplantation due to their definitive and accurate phasing of the individual polymorphisms of key SNPs across the region. However these methods are very time consuming and not practical for large cohorts of individuals. One new emerging technology that can be applied to achieve HLA typing is next generation sequencing (NGS) [12], [49], [50]. Such technologies enabling single molecule sequencing in high throughput are becoming more widely available for HLA typing with more accurate computational tools [51] and implementation in clinical HLA laboratories [52], [53]. However, accurate phasing is reliant on generating large enough fragment sizes which is currently limiting. In addition, although prices are becoming more competitive, to sequence cohorts of individuals is still prohibitively expensive. Imputation, as presented here, although not meant to replace typing methods for the clinical environment, represents a robust complementary approach applicable to the research community for very little additional cost that can maximize the value of existing high density SNP array data currently available on many cohorts around the world.

## Figures and Tables

**Table 1:** Summary of imputed HLA alleles for OBB British Caucasian population (n = 5553).

	Total number of different imputed two-field resolution alleles	% individuals with imputed with a high certainty (posterior probability >0.95)
HLA-A*	32	90.89%
HLA-C*	22	94.06%
HLA-B*	56	88.40%
HLA-DRB1*	34	67.42%
HLA-DQA1*	8	98.60%
HLA-DQB1*	16	95.26%
HLA-DPA1*	6	96.42%
HLA-DPB1*	21	72.48%

**Table 2:** Imputed amino acid polymorphisms for 8 HLA loci in OBB British Caucasian population (n = 5544). The numbers refer to the amino acid positions identified as polymorphic within the protein sequence.

	Total number of polymorphic positions	Total number amino acid substitutions imputed at those positions	Number of bi-allelic positions	Number of multi-allelic positions
HLA-A*	78	208	35	43
HLA-C*	64	129	48	16
HLA-B*	74	237	50	24
HLA-DRB1*	51	255	13	38
HLA-DQA1*	36	59	28	8
HLA-DQB1*	53	159	15	38
HLA-DPA1*	15	15	15	0
HLA-DPB1*	22	46	10	12

Total	393	1108	214	179
