# Supplementary material for: High resolution HLA haplotyping by imputation for a British population bioresource
Source: Hum Immunol. 2017 Mar;78(3):242–51. doi: 10.1016/j.humimm.2017.01.006 (PMC5367449; doi:10.1016/j.humimm.2017.01.006)
Supplement: Supplementary data 1 [file mmc1.pdf]

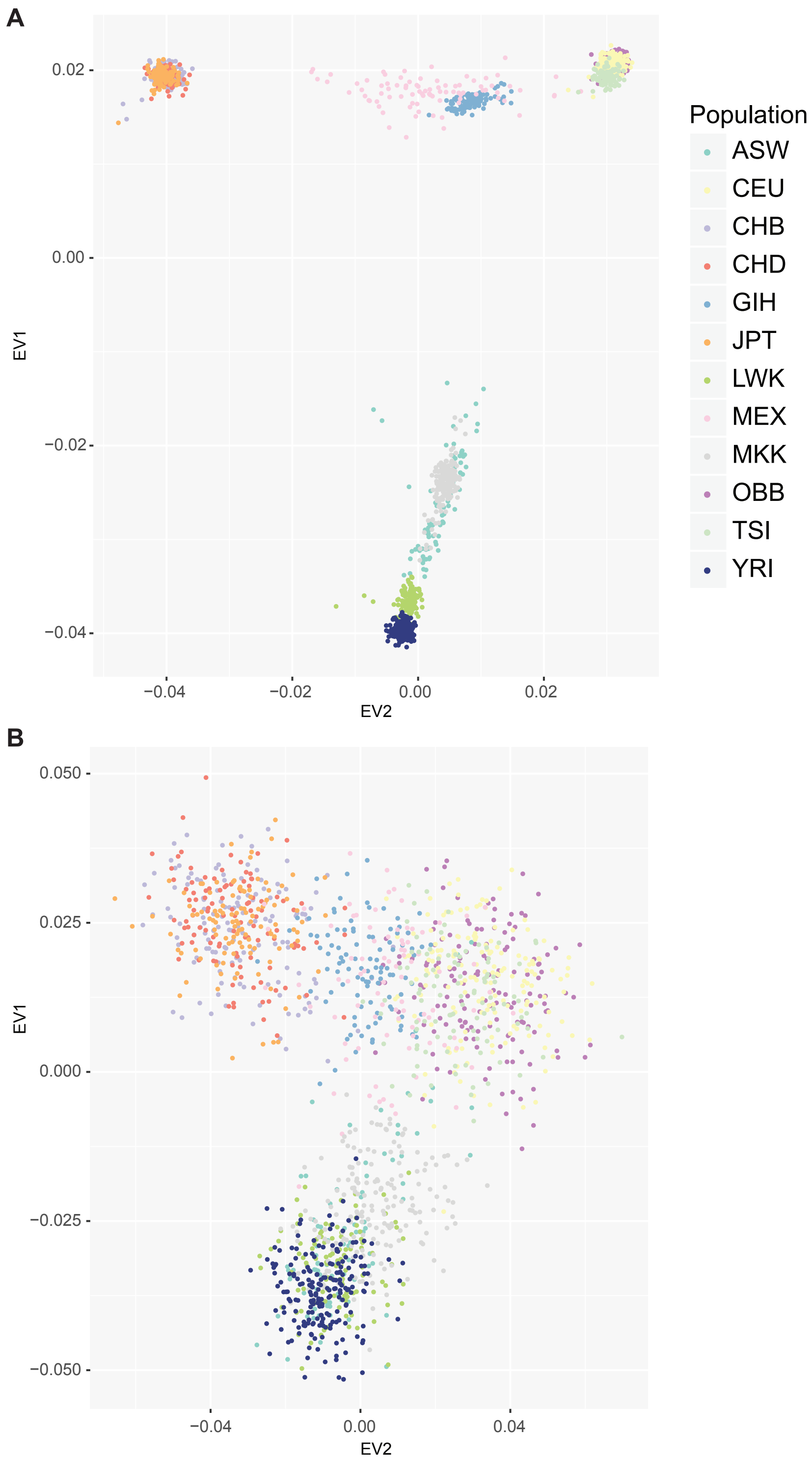

Supplementary figure 1. Principal component analysis comparing Oxford Biobank participants with 11 different human populations from the HapMap project. (A) Data is shown for 150 randomly selected Oxford Biobank participants to ensure comparable sample size to the HapMap populations. First and second principal components shown (plotted as eigenvectors, EV). OBB (Oxford biobank, n=150); ASW (African ancestry in Southwest USA, 87); CEU (Utah residents with Northern and Western European ancestry from the CEPH collection, 165); CHB (Han Chinese in Beijing China, 137); CHD (Chinese in Metropolitan Denver Colorado, 109); GIH (Gujarati Indians in Houston Texas, 101); JPT (Japanese in Tokyo Japan 113); LWK (Luhya in Webuye Kenya 110), MEX (Mexican ancestry in Los Angeles, California 86); MKK (Maasai in Kinyawa Kenya 184); TSI (Toscani in Italia, 102); YRI (Yoruba in Ibadan Nigeria, 203). (B) PCA restricted to MHC region.
